# BME2.1: The Need for a Systems Approach to Addressing Race-Based Disparities in Health and Health Care

**DOI:** 10.34133/bmef.0023

**Published:** 2023-06-21

**Authors:** Naomi C. Chesler, Gilda A. Barabino

**Affiliations:** ^1^ Edwards Lifesciences Foundation Cardiovascular Innovation and Research Center and Department of Biomedical Engineering, Samueli School of Engineering, University of California, Irvine, CA, USA.; ^2^ Olin College of Engineering, Needham, MA, USA.

The recent article by Miller et al. [1] in *BMEF* on reimagining the Biomedical Engineering (BME) curriculum of the future proposes several exciting and transformative core principles. These include (a) incorporating modern molecular biology and analytical/computational modeling, (b) providing instruction in data science fundamentals, (c) integrating clinical needs for innovation and translation, (d) fostering an educational culture of inclusive excellence, and (e) ensuring that new research discoveries inform the core curriculum.

This vision of BME2.0 is motivated in part by an “industrial revolution” in artificial intelligence and machine learning [2] but was constructed with little attention to the societal revolution that began with the launch of the Black Lives Matter movement (by Alicia Garza, Patrisse Cullors, and Opal Tometi, as a response to the killing of Black people by vigilantes and especially police) and grew into nationwide protests with the videotaped killing of George Floyd. For many, increased recognition of the ways in which racially biased vigilantism and police violence dramatically cut short Black lives in our country has been accompanied by awareness of a racial pandemic in health and health care [3] that reduces both lifespan and quality of life for our Black citizens. With this motivation, any transformation of the BME curriculum not only should “champion an educational culture of inclusive excellence” [1] but also must include education and training in race-based disparities in health and health care to enable our BME graduates to contribute to a better future for all.

The distinction between health disparities and health care disparities is important. Heath equity, defined by the Centers for Disease Control and Prevention as “the state in which everyone has a fair and just opportunity to attain their highest level of health” [4], depends on social determinants of health (SDOH), which are the non-medical factors that influence health outcomes. According to the World Health Organization, these are “the conditions in which people are born, grow, work, live, and age, and the wider set of forces and systems shaping the conditions of daily life” [5]. Evidence of race-based health inequity has been accumulating over the last 2 decades [6]. For example, Black Americans are at increased risk for hypertension, obesity, and diabetes compared to White Americans, and these disparities contribute to the burden of cardiovascular disease (CVD) [7]. Race-based disparities are compounded by sex-based disparities, with Black women suffering from more CVD risk factors, developing CVD earlier, and having higher CVD mortality rates than White women [8]. Moreover, members of the LGBTQ+ community who are Black have higher CVD risk factors than those who are not [9]. This heavier burden and earlier onset of poor health outcomes observed in Black Americans are not fully explained by other sociodemographic factors (e.g., age and education). While some small part of these disparities may be related to ancestry (i.e., genetics), the evidence is clear that the social construct of race and its consequence, racism, are the dominant contributors [10–13]. In as much as racism in US society is immutable, the impact of racism on the health of Black and African Americans can be considered a non-modifiable factor in research problem identification, the knowledge of which should inform experimental design, research project implementation, and ultimately engineering solutions. As BME2.0 students solve real-world problems with clinical impact, solutions that address, reduce, and eliminate health disparities are critical, especially for the Black community and other minoritized and socially disadvantaged populations.

Health equity also depends on access to health care, and even when access to providers is equitable (though it generally is not), bias in medical technologies, devices, and data interpretation by algorithms and providers can introduce health care inequities that are simultaneously insidious and perilous. For example, researchers at Emory University found that there was a 26% lower chance of detecting a fever in hospitalized Black individuals when health workers used forehead thermometers compared to oral thermometers [14]. There was no discrepancy for White patients, suggesting that the technology used to detect a fever is sensitive to skin tone. According to the authors of the study, “differences in detection of fever could lead to delays in antibiotics and medical care for Black patients", which could contribute to increased death rates among Black patients. The motivation for this investigation was an earlier study by researchers at the University of Michigan Hospital, which found that pulse oximeters overestimate blood oxygen levels in patients who have darker skin, potentially delaying necessary treatment and putting patients at risk [15]. Remarkably, the effect of skin pigmentation on pulse oximeter accuracy was first reported 15 years earlier [16], but neither the public nor the medical establishment took enough notice to either change care practices or force redesign of these ubiquitous medical devices.

When the devices themselves do not introduce bias, guidelines for interpretation can do so. Based on beliefs that Black people have denser bones, more muscle, or thicker skin, Black patients were routinely exposed to higher doses of radiation for x-rays than White patients from the 1860s to the 1960s, when a public report [17], senate hearings, and a public outcry ended the practice [18]. Similarly, pulmonary function test results have historically been interpreted with race-specific equations for expected values that lead to undertreatment of lung disease in people of color; still, use of these equations remain a matter of debate among professionals [19]. Even electronic medical records have been found to introduce race bias into health care. A study in *Science* concluded that an algorithm used to manage care for 200 million people in the US referred fewer equally sick Black people for programs to improve care for patients with complex medical needs than White people [20]. Since biomedical engineers play an important role in the development of these technologies, devices, and algorithms, their education and training in this domain are imperative.

As highlighted by the recent perspective article by Barabino and Nembhard [3], engineering education regarding bias and racism in health (impact of race and SDOH on health and disease) and health care (access as well as medical devices, technologies, and algorithms) is critical to addressing the current racial pandemic in health and health care. Indeed, these changes in engineering education are key components of a comprehensive, systems approach to addressing disparities in health and health care, along with government policies to reduce bias and racism in public health, equity-driven health care markets, and broader access to medical treatments. Therefore, we suggest fundamentals of sex as a biological variable and race as a societal variable with biological consequences in any presentation of modern molecular biology and analytical/computational modeling. We also recommend providing instruction in inclusive study design within the context of data science fundamentals. Similarly, innovation and translation must begin with needs-finding among diverse populations, include testing on heterogeneous groups, and develop translation strategies that increase, and do not decrease, access.

Thus, we applaud the transformative vision of BME2.0 and offer additional guidance for programs to incorporate the current reality of racial bias and its impacts, including race-based health and health care disparities. We urge BME education and training programs to work to impart a comprehensive and collective understanding that race-blind engineering solutions, especially those created by and tested on homogeneous populations, can cause serious harm. Further, engineering solutions to problems that only affect a narrow subset of the population can worsen health disparities. Finally, engineering solutions that do not consider cost, complexity, and insurance coverage will worsen health care disparities. Instead, by weaving health and health care equity topics throughout all aspects of BME education using a systems approach, we will not only generate better medical devices, technologies, and algorithms, we will also create a future generation of problem-solvers and critical thinkers who are equipped to tackle our society’s grand challenges, including health and health care equity.

## References

[1] Miller MI, Brightman AO, Epstein FH, Grande-Allen KJ, Green JJ, Haase E, Laurencin CT, Logsdon E, Gabhann FM, Ogle B, et al. BME 2.0: Engineering the future of medicine. BME Front. 2023;4:0001. 10.34133/bmef.0001 PMC10530648 37849657

[2] Butt A. AI is the fourth industrial revolution technology. 18 Nov 2019. [accessed 27 Feb 2023] https://readwrite.com/ai-is-the-fourth-industrial-revolution-technology/.

[3] Barabino GA, Nembhard HB. Suffocating from medical bias: A systems engineering approach to equitable health care solutions. Am Sci. 2022;110(4):204.

[4] Centers for Disease Control and Prevention. What is health equity? 1 Jul 2022. [accessed 27 Feb 2023] https://www.cdc.gov/healthequity/whatis/index.html

[5] World Health Organization. Social determinants of health. [accessed 27 Feb 2023] https://www.who.int/health-topics/social-determinants-of-health#tab=tab_1

[6] Williams DR, Lawrence JA, Davis BA, Vu C. Understanding how discrimination can affect health. Health Serv Res. 2019;54(Suppl 2):1374–1388. 31663121 10.1111/1475-6773.13222 PMC6864381

[7] Mouton CP, Hayden M, Southerland JH. Cardiovascular health disparities in underserved populations. Prim Care. 2017;44(1):e37–e71. 28164826 10.1016/j.pop.2016.09.019

[8] Williams RA. Cardiovascular disease in African American women: A health care disparities issue. J Natl Med Assoc. 2009;101(6):536–540. 19585921 10.1016/s0027-9684(15)30938-x

[9] Caceres BA, Brody A, Luscombe RE, Primiano JE, Marusca P, Sitts EM, Chyun D. A systematic review of cardiovascular disease in sexual minorities. Am J Public Health. 2017;107(4):e13–e21. 10.2105/AJPH.2016.303630 PMC5343694 28207331

[10] Chae DH, Clouston S, Martz CD, Hatzenbuehler ML, Cooper HLF, Turpin R, Stephens-Davidowitz S, Kramer MR. Area racism and birth outcomes among Blacks in the United States. Soc Sci Med. 2018;199:49–55. 28454665 10.1016/j.socscimed.2017.04.019 PMC5640467

[11] Chae DH, Martz CD, Fuller-Rowell TE, Spears EC, Smith TTG, Hunter EA, Drenkard C, Lim SS. Racial discrimination, disease activity, and organ damage: The Black women's experiences living with lupus (BeWELL) study. Am J Epidemiol. 2019;188(8):1434–1443. 31062841 10.1093/aje/kwz105 PMC6670046

[12] Leitner JB, Hehman E, Ayduk O, Mendoza-Denton R. Blacks' death rate due to circulatory diseases is positively related to whites' explicit racial bias. Psychol Sci. 2016;27(10):1299–1311. 27557618 10.1177/0956797616658450

[13] Paradies Y, Ben J, Denson N, Elias A, Priest N, Pieterse A, Gupta A, Kelaher M, Gee G. Racism as a determinant of health: A systematic review and meta-analysis. PLOS ONE. 2015;10(9): e0138511. 26398658 10.1371/journal.pone.0138511 PMC4580597

[14] Bhavani SV, Wiley Z, Verhoef PA, Coopersmith CM, Ofotokun I. Racial differences in detection of fever using temporal vs oral temperature measurements in hospitalized patients. JAMA. 2022;328(9):885–886. 36066526 10.1001/jama.2022.12290 PMC9449792

[15] Sjoding MW, Dickson RP, Iwashyna TJ, Gay SE, Valley TS. Racial bias in pulse oximetry measurement. N Engl J Med. 2020;383(25):2477–2478. 33326721 10.1056/NEJMc2029240 PMC7808260

[16] Bickler PE, Feiner JR, Severinghaus JW. Effects of skin pigmentation on pulse oximeter accuracy at low saturation. Anesthesiology. 2005;102(4):715–719. 15791098 10.1097/00000542-200504000-00004

[17] Mintz NY. State health department orders change in negro x-ray use. *Washington Post*. 1968;A3.

[18] Bavli I, Jones DS. Race correction and the x-ray machine—The controversy over increased radiation doses for Black Americans in 1968. N Engl J Med. 2022;387(10):947–952. 36069880 10.1056/NEJMms2206281

[19] Bhakta NR, Kaminsky DA, Bime C, Thakur N, Hall GL, McCormack MC, Stanojevic S. Addressing race in pulmonary function testing by aligning intent and evidence with practice and perception. Chest. 2022;161(1):288–297. 34437887 10.1016/j.chest.2021.08.053 PMC8783030

[20] Obermeyer Z, Powers B, Vogeli C, Mullainathan S. Dissecting racial bias in an algorithm used to manage the health of populations. Science. 2019;366(6464):447–453. 31649194 10.1126/science.aax2342

